# Interaction of Microbes with Microplastics and Nanoplastics in the Agroecosystems—Impact on Antimicrobial Resistance

**DOI:** 10.3390/pathogens12070888

**Published:** 2023-06-29

**Authors:** Jayashree Nath, Jayita De, Shantanu Sur, Pratik Banerjee

**Affiliations:** 1 Department of Food Science and Human Nutrition, University of Illinois at Urbana-Champaign, Urbana, IL 61801, USA; 2 Department of Biology, Clarkson University, Potsdam, NY 13699, USA

**Keywords:** microplastics, nanoplastics, antimicrobial resistance, horizontal gene transfer, agroecosystems

## Abstract

Microplastics (MPs) and nanoplastics (NPs) are hotspots for the exchange of antimicrobial resistance genes (ARGs) between different bacterial taxa in the environment. Propagation of antimicrobial resistance (AMR) is a global public health issue that needs special attention concerning horizontal gene transfer (HGT) under micro-nano plastics (MNPs) pressure. Interactions between MNPs and microbes, or mere persistence of MNPs in the environment (either water or soil), influence microbial gene expressions, affecting autochthonous microbiomes, their resistomes, and the overall ecosystem. The adsorption of a range of co-contaminants on MNPs leads to the increased interaction of pollutants with microbes resulting in changes in AMR, virulence, toxin production, etc. However, accurately estimating the extent of MNP infestation in agroecosystems remains challenging. The main limitation in estimating the level of MNPs contamination in agroecosystems, surface and subsurface waters, or sediments is the lack of standardized protocols for extraction of MPs and analytical detection methods from complex high organic content matrices. Nonetheless, recent advances in MPs detection from complex matrices with high organic matter content are highly promising. This review aims to provide an overview of relevant information available to date and summarize the already existing knowledge about the mechanisms of MNP-microbe interactions including the different factors with influence on HGT and AMR. In-depth knowledge of the enhanced ARGs propagation in the environment under the influence of MNPs could raise the needed awareness, about future consequences and emergence of multidrug-resistant bacteria.

## 1. Introduction

The term “microplastics” (MPs) was first coined by Thompson in 2004 to describe the small (less than 5 mm in size) plastic fragments in the oceans [1]. Since then, MPs from almost all parts of the ecosystem have been reported extensively, indicating possible deleterious consequences on living systems. The early research on MPs focused more on the estimation of the levels of contamination in the ecosystem and understanding the fate of such plastic components of different sizes, shapes, or types in marine and terrestrial animal tissues [2,3,4]. A growing number of recent studies explain the physiological threat posed by MPs [5,6,7]. With the gradual infestation of agricultural soil by MPs, the impact of MPs on agroecosystems [8,9], groundwater resources [10,11], bioaccumulation of MPs in plants, and trophic transfer to the food chain via food crops is gaining increased attention [12]. MPs in aqueous environments or agroecosystems can easily migrate throughout the ecosystem, causing more complex environmental problems. Reports show that MPs in agricultural soil could affect soil pH, respiration, and enzymatic activities depending on MP shape and polymer type [13]. Some recent articles about MPs in agroecosystems [14,15,16,17] shed light on many aspects such as sources of MPs, their transformation, transport, effects to agricultural soils, etc., which have raised concerns about persistence of MNPs in ecosystem and a range of their negative effects such as other anthropogenic pollutants, such as changes in microbial community structure, resistome, and horizontal gene transfer (HGT), which are discussed in this review. Plastic materials have become essential agronomic components for farmers and growers and are used in a wide range of applications as presented in Table 1. Consequently, the route of entry for MPs in agroecosystems are through agricultural fertilizers, agricultural plastic film mulch, sewage/irrigation pipes, etc., and sometimes accidental release of MPs, e.g., through the application of sewage sludge. However, upon long-term use, such plastic materials undergo aging and degradation mostly due to ultraviolet light or other environmental factors such as rain, storm, and wind that accelerate the formation of MPs [9]. Nanoplastics (NPs) are of sizes less than 100 nm, which typically originate as a result of further degradation of MPs and are more interactive with living systems due to their extremely small sizes and larger surface areas [18]. Primary sources of micro-nanoplastics (MNPs) are the micro- or nano-sized particles frequently utilized in biomedical and several other consumer application products, while secondary source of MPs results from unregulated disposal of macro-plastic wastes and their subsequent physicochemical and biological degradation in water or soil. Over several years of transformed agricultural practices, plastic components such as protective cultivation films, irrigation/drainage pipes, or protective nets, etc., have helped farmers boost crop productivity while minimizing operational costs, and have become essential agronomic components [19].

**Figure 1:**
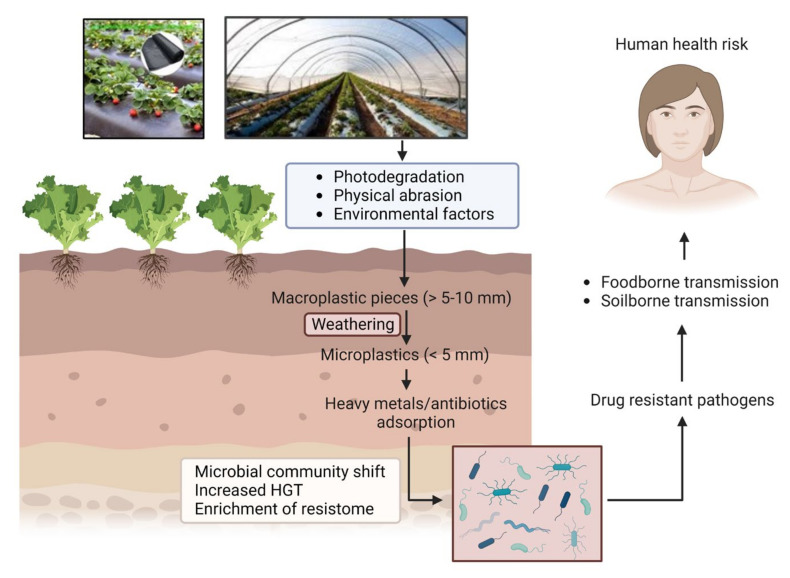
Pathway of MP/NP mediated antimicrobial resistance through agroecosystem.

Several recent studies describe the interactions between MPs and microbes in soil and aqueous environments [21,22,23]. Microbes in the environment aggregate on different organic or inorganic surfaces, such as natural environmental components, including leaves or rocks, etc. In addition, as emerging anthropogenic contaminants, MPs are found to act as excellent abiotic surface for attachment of microbial communities. Consequently, MPs in terrestrial and aquatic ecosystems can drive changes in the whole microbiome structure and may modulate different essential nutrient and biogeochemical cycles in the ecosystem [24,25]. Environmental infestation by MPs has been implicated with another critical public health problem—antimicrobial resistance (AMR). AMR has emerged as a global challenge for human health, agriculture, and animal husbandry. Although resistance development is a multifaceted problem, the environment plays a key role in facilitating resistance evolution and transmission between bacterial species. One major concern for the environmental contamination with MPs is its potential to accelerate microbial interactions and activities by providing suitable surface for attachment of microbes, especially pathogens. Such a process can be driven by a number of mechanisms acting alone or in combination [26]. One such mechanism is attributed to the high surface area to volume ratios provided by MPs, allowing multiple bacterial species to attach and grow, eventually leading to biofilm formation [27]; compared with the surrounding environment, bacterial communities (BC) in these “plastisphere” cohabit in close proximity and at higher density, enabling a more efficient exchange of genetic materials. In certain situations, the enrichment of antimicrobial resistance genes (ARGs) and mobile genetic elements (MGEs) in MP biofilms may facilitate the HGT mechanisms [28]. Moreover, the adsorption of antibiotics and heavy metals on MP surfaces can expose BCs to higher concentrations of these factors, leading to cross-resistance (resistance to antibiotics and metals) or co-resistance (resistance to multiple antibiotics) [29]. Thus, antimicrobial resistance genes (ARGs) and MNPs both are emerging contaminants in the environment, which are of great public concern due to their potential risk to human health. Figure 1 depicts the route of entry of MPs into the agricultural soil and how it ultimately becomes a health hazard. The aim of this review is to synthesize available information relevant to understanding the interaction of MNPs with the microbiome in agroecosystems affecting microbial resistome and their role in the enhancement of AMR propagation. This is an important issue that came into focus recently and deserved immediate attention to prevent future complications. This review aims to fill in the knowledge gaps and highlight key future research areas in this field with special attention to the factors enhancing the interactions between MNPs, environmental ARGs, and microbes in the soil.

## 2. The Physiologic Implications of Microbe–MNP Interactions

### 2.1. MNP-Induced Gene Expressions and Gene Transfers

Microorganisms stimulate multiple physiological responses in the presence of MNPs, such as enhanced production of intracellular reactive oxygen species (ROS), increased membrane permeability, and enhanced expression of certain genes. Physical and environmental factors such as size of MNPs, UV-aging, and leachates from MNP degradation are reported for stimulations of such reactions in microbes [30,31,32]. MNPs, especially ~100 nm or smaller, can interact with the microbial surface receptors causing structural changes in the membrane, which allows the internalization of MNPs through endocytosis and ROS generation by NADPH oxidase [33,34]. A stronger interaction may lead to higher ROS production; however, the viability of both recipient bacteria and donor bacteria decreases significantly with the overproduction of ROS. Higher gene transfer efficiency due to increased ROS response are reported unless the ROS production was so high that it caused excessive damage to bacteria [33,35]. MNPs have been reported to increase the expressions of genes related to conjugation (*trbBp* and *traF*), global regulatory genes (*korA*, *korB*, and *trbA*), outer membrane genes (*ompA* and *ompC*), etc. Recent transcriptomics studies showed up-regulation of genes controlling ARG conjugation (*kilA* and *traI*), cell permeability (*ompR*), and intracellular ROS production (*recA*) upon exposure to aged MPs relative to pristine MP exposures, contributing to enhanced HGT [5,30,33]. All these studies indicated conjugation and transduction as the dominant mechanism for HGT [36].

### 2.2. MP-Associated Biofilms and Cluster Formation

MPs existing in several different forms, such as fibers, films, foams, or pellets, may serve as effective abiotic surfaces for microbial colonization due to their small size but bigger surface area, surface texture, hydrophobic composition, and long persistence. Recent studies have detected different forms of plastics and measured more biomass formation on MP-biofilm than on rock surfaces in river/lake environments [27,37,38]. Plastics-associated microbial communities were found to be distinctly different from those on the non-plastic substrates [39]. Biofilm formation is known to be a dynamic process, which generally involves microbial adhesion, followed by the secretion of extracellular polymeric substances (EPS) and then microbial proliferation. Microbiota associated with MPs, over time, become the platform for association of other microbes and pathogens, which many times facilitates interaction and transfer of antimicrobial resistance genes (ARGs) among microbes. The expression of quorum-sensing genes by the bacteria accumulated on a surface is also reported to cause further cell-to-cell adhesion, maturation, and dispersion of biofilms [40]. Quorum sensing also plays a crucial role in microbial communication that helps in the initial attachment of microbes to the MP surface and further proliferation of biofilm formation, leading to increased bacterial resistance. The EPS secreted by adhering microbes on MPs is a viscous layer that enhances the adsorption of many environmental pollutants, such as toxic organic compounds, xenobiotic compounds, heavy metals, etc. [41]. The formation of biofilms on MPs depends on several physical and chemical properties of plastic substrates, such as polymer type, surface properties, and particle sizes. For example, polyvinylchloride (PVC) and polyethylene (PE) MPs favored enhanced bacterial adhesion more than polypropylene (PP) and polyethylene terephthalate (PET) MPs due to their larger specific surface area and roughness [42]. The surface properties of MPs significantly influence the colonization by bacterial cells on them. Some studies indicate that differences in the attachment and compositions of bacterial communities were surface-hydrophobicity-dependent on MPs [39,43]. Surface charges and sizes of MPs and MNPs also influence microbial attachment and biofilm formations, which are discussed in the following sections of this paper. The growth of biofilm on MPs changes their density, influencing their mobility in the environment (water or soil), or often leads to partial degradation, thereby enhance the release of leachates and toxic substances into the environment. Bacteria associated with biofilms often create defense colonies against antimicrobial agents or harsh environments for their survival, which helps in the spread of AMR [44]. Certain biofilm-associated microbes, such as *Pseudomonas aeruginosa*, are reported to show increased expression levels of various efflux pumps leading to the release of resistance genes into the environment and enhanced AMR [45]. Metagenomic sequencing and meta-transcriptomic sequencing methods reveal the enhanced transcriptional activity of several ARGs in MP-associated biofilms or plastisphere [46]. The role of plastic types was elucidated, such as PET plastics associated with biofilm were found to influence the ARG composition significantly [47]. Encapsulation of biofilms or colonizing of microbes on MP surfaces acts as a reservoir of microbial pathogens and becomes the hotspot for HGT among the biofilm-forming microbes or other microbes through various MGEs, ARGs [48].

## 3. Influence of MNPs in the Propagation of AMR in Agroecosystem

Recent studies have highlighted the role of MNPs in enhancing the mobility and transfer of ARGs and metal resistance genes (MRGs) in the ecosystem, causing the enhanced spread of AMR. Any agent or situation promoting AMR is considered a threat to global public health. A growing body of scientific evidence correlates the elevation of AMR indicators (i.e., HGT, the abundance of ARGs, MGEs) in the presence of persistent MNPs in an ecosystem. However, elaborated information on underlying mechanisms and experimental evidence on the interaction of MNPs with microbes causing the propagation of AMR is still naive. Table 2 summarizes some of the recent research papers (with a brief description of the work done and main findings) updating the current state of knowledge in this area. The conjugative transfer or HGT of antibiotic resistance genes in *E. coli* are reported to be highly dependent on the size of MPs [33]. UV-aged polystyrene microplastics (PS-MPs) were found to increase the HGT of ARGs in *E. coli* [30]. MP/NPs were reported to promote the propagation of ARGs in phosphorus-removing bacteria and induced microbial community shift [49]. MPs also demonstrated selective inhibition of ammonia-oxidizing bacteria and enrichment of nitrite-oxidizing bacteria, leading to partial nitrification [35]. These publications reported similar effects in bacterial systems upon exposure to MPs, such as increased ROS generation, increased cell permeability, and upregulation of HGT-associated genes.

### 3.1. Impacts on Resistome

The reports on the interaction of MNPs and microbial communities to date strongly show that MPs could highly influence the microbial resistome or changes in the resistome of environmental niches. Several other reports provide evidence that changes in the resistome profile of different groups of microbes due to the presence of MNPs could further enhance the propagation of AMR genes in the environmental compartments as a whole [51,52].

#### 3.1.1. In the Wastewater Treatment System

Several studies have reported changes in resistome of wastewater sludge systems (which contain high content of ARGs, MNPs, and other contaminants. Studies reported shifts in microbial community structure and phosphorus removal efficiency of wastewater effluent treatment systems, under MPs pressure [49]. The sludge community structure, detected by high-throughput sequencing technology, showed that the abundance of dominant phyla was considerably shifted in the presence of MPs, which led to profound changes in resistome. Other groups of microbes replaced the more abundant polyphosphate accumulating organisms in sludge; however, the phosphorus removal efficiency was not significantly affected. Similarly, the effects of MPs on the nitrification of aerobic granular sludge (AGS) and shifts in the microbial communities due to an increased abundance of intracellular and extracellular ARGs are reported [35]. The relative abundance of *Nitrosomonas* decreased significantly, while the abundance of *Nitrospira* and other nitrifying bacteria showed an increase. In another study, microbial community composition and abundance of ARGs and MRGs on the plastisphere borne biofilm and planktonic bacteria in wastewater treatment plant (by qPCR) revealed a higher abundance of potentially pathogenic bacteria in treated wastewater on the plastisphere than in the planktonic bacterial community [53].

#### 3.1.2. In the Water Ecosystem

MPs in aquatic bodies provide bacterial niches, which serve as hotspot for dissemination of ARGs, and influence the resistome. A clear variation for ARG profiles was elucidated through metagenomic studies between river water and MPs from the water [54]. Some special ARGs were detected in MPs-associated biofilm, but not in the surrounding river water, indicating selective enrichment and changes in resistome [27]. Twelve ARGs/MGEs, including four genes responsible for resistance to tetracycline and ampicillin were detected in sewage water in a study, which provided significant information about the combined disposal of MPs, ARGs, and pharmaceuticals in domestic sewage [55]. Comparison between bacterial taxa, specially pathogenic bacteria and ARGs, on the plastisphere and water revealed the extensive influence of urbanization on the selectivity of microbes and ARGs for MPs [56]. Metagenomic insights into the ARGs and their hosts on biodegradable and non-biodegradable PET-based MPs suggested selective preferences for two types of plastics harboring ARGs and influencing the resistome in a coastal lagoon in the northern Gulf of Mexico [47].

#### 3.1.3. In Soil

Soil is the largest reservoir for several environmental contaminants, which include antibiotic-resistant genes or other mobile genetic elements. Selective enrichment of ARGs or specific groups of bacteria, particularly pathogenic microbes on plastisphere, may increase or contribute more to the pathogenicity by antibiotic resistance gene transfer, making it difficult to kill pathogens that have acquired the ARGs. Overall higher relative abundances of 102 ARGs and 3 MGEs are observed in the plastisphere present in soil compared to the soil environment itself through DNA extraction from soil and sequencing [23]. Similarly, soil DNA extraction followed by high-throughput sequencing showed a significantly higher number of total ARGs in the plastisphere compared to the surrounding soil for all MP types and sizes considered in the study [5]. Higher ARGs abundance on larger and aged MPs in the soil after ten years of crop planting is detected using a high-throughput fluorescence quantitative reaction platform [57]. The changing patterns of ARGs and the bacterial communities on different types and sizes of MPs in soil environments through metagenomic approaches are reported [6].

### 3.2. Impacts on the HGT

**Figure 2:**
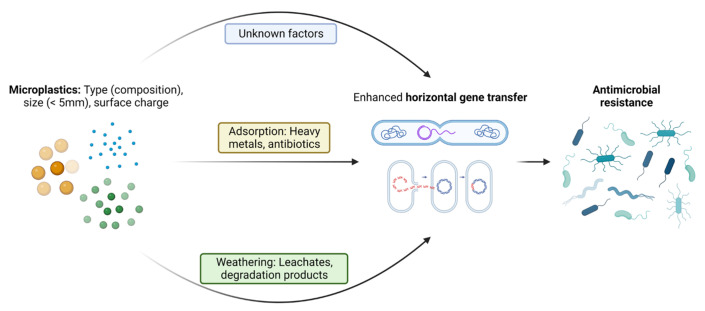
Pictorial representation of the crucial factors in MNPs-mediated enhancement of the horizontal transfer of intracellular and extracellular antibiotic-resistant genes among microbes.

Factors influencing the dissemination and proliferation of ARGs in the presence of MPs have come into focus recently. The surrounding environment has been considered an important factor influencing the attached communities compared to the polymer type [58]. Specific bacterial assemblages are also now known to play a crucial role in ARGs’ persistence and proliferation on the plastisphere, since close and stable relations between ARGs and bacterial taxa have been identified [59]. The abundance of antibiotics in the environment due to misuse of antibiotics is another important factor for the selection and persistence of ARGs in the environment [13]. The leachates from MP degradation in soil or due to natural aging may exert additional pressure on enhancement of AMR in opportunistic pathogens over a long time span. As also reported by [23], the presence of a range of potential pathogens and ARGs/MGEs in the plastisphere enhances a high risk of ARGs entering potential pathogens. Although it is not clear whether the residual antibiotic drives the selection of the associated resistant gene, it is more likely that antibiotic pressure contributes to the emergence of co -resistance since some types of ARGs may be co-selected [60]. Some specific factors regarding the interaction of MPs and microbes that have been reported to the date to be responsible for proliferation of AMR are discussed in more detail below. Figure 2 shows a pictorial representation of the factors leading to enhanced ARGs transfer into pathogenic bacteria.

#### 3.2.1. Size of MPs

As listed in Table 2, the influence of MPs on gene transfer efficiency is reported to be dependent on their sizes [33]. The relationship between the size distribution of MPs and its impact on gene transfer is an important factor and must be understood in detail. In previous study, smaller MPs (<50 nm) were reported to increase the gene transfer; however, at very high concentrations (>100 mg/L), they exert a negative effect on cell viability and the mechanism of gene transfer [33]. Moderate-sized MPs (~100 nm) increased the gene transfer efficiency significantly with an increased concentration of MPs. Large MPs (>1 µm) caused no significant increase in gene transfer efficiency [33]. It is to be noted that the large plastic particles (hundreds of micrometers or at least >5 µm) provide a broad aggregation surface for bacteria to grow on, which in many publications has been referred to as plastisphere. When different types of bacteria attach to the plastisphere surface, the contact between those bacteria becomes more stable, which ultimately results in increased gene transfer efficiency. Several researchers have demonstrated that ARGs can also adsorb directly on the surface of extremely large MPs (hundreds of micrometer scales), accelerating the dissemination of antibiotic-resistance genes in the environment [16,47]. However, for MPs of sizes smaller than bacteria or near about similar, it is impossible for bacteria to grow on the MPs surface, and such MPs could enhance the gene transfer by inducing more ROS production and increasing cell membrane permeability. The large MPs do not influence the gene transfer because they fail to change these factors and just support stable contact between microbes with MPs and many other contaminants such as heavy metals. Increase of tetracycline resistance gene abundances under MP pressure was found higher than under NP pressure; this difference is potentially due to a stronger selective pressure from the inhibition of growth and metabolism of resistant microorganisms by NPs, resulting from more leaching of chemical additives and increased ease to pass-through biological barriers [49].

#### 3.2.2. Type and Concentration of MPs

The nitrification performance of the aerobic granular sludge (AGS) was inhibited by different concentrations and different types of MPs [35]. Different types of MPs, such as PVC, PE, polyamide (PA), and polystyrene (PS), differentially affected the ammonia removal efficiency and the nitrification function of AGS. Furthermore, the concentration of MPs influenced the nitrification function of AGS, with the nitrification inhibited upon increasing the concentration from 1.0 mg/L to 10 mg/L. Accordingly, nitrification-function-related genes were found to be affected differently by different concentrations of MPs. Although bio-adhesion was observed on the surface of all MPs, it was more pronounced on PE [33]. The lowest MP concentration at which an effect on gene transfer efficiency is observed is ~0.1 mg/L; however, the maximum concentration is limited by the low solubility of MPs as very high concentration of MPs may cause technical problems, such as causing flocculent precipitates and may interfere with results [33]. Therefore, 100 mg/L was usually set as the upper limit for the concentration of MPs in most reported studies.

#### 3.2.3. Aging of MPs

As listed in Table 2, the UV-aged MPs, obtained by exposing MPs to UV for up to 20 days, were found to increase the gene transfer frequency relative to the pristine MP without any treatment [30]. UV irradiation was found to substantially alter the surface chemistry and morphology of MPs, like the natural aging process. For pristine non-rinsed MPs, HGT enhancement was mainly caused by proximal adsorption of ARG donors and recipients, while the contribution from the leachates from MPs was marginal. For aged non-rinsed MPs, both adsorption and leachate contributed to the enhanced ARG transfer frequency, and the latter became more predominant with increased aging. Leachates from aged MPs exert higher oxidative stress on bacteria, thus inducing more intracellular ROS and an increase in cell permeability than those from pristine MPs. While pristine MPs interact with biomolecules mainly via hydrophobic attraction and π-π stacking, the aged MPs with more oxygen-containing functional groups (e.g., C–O-OH) on the surface may form more stable hydrogen bonds with the ARG vectors. A stronger interactive force contributed to the enhanced adsorption capacity of non-rinsed UV-aged MPs towards both ARG donors and recipients. Along with increased retention of ARG donors and recipients, they also increased cell permeability relative to unexposed controls. All these factors contribute to the enhancement of HGT.

## 4. Impacts of Long-Term Persistence of MPs on Agroecosystem

The persistence of MNPs in an ecosystem can disrupt the delicate balance that exists between its various components. In agroecosystems, MNPs can significantly affect the soil microbial biomass, microbial activity, and functional diversity [61]. MNPs can also alter the essential nutrient cycles (plant nutrient elements distribution), which are reported to affect plant seed germination or seedling growth indirectly [62]. Additionally, MPs can block some of the important initial plant life-cycle functions, such as gas exchange and water absorption, along with a potential impact on soil functions by changing the interactions between soil organisms and the surrounding environment. MPs that persist in agroecosystems for a long period gradually undergo aging, which involves leaching out of additives/plasticizers and eventually breaking down into NPs. Various factors, including environmental effects, photodegradation, and/or soil microbial enzyme-mediated degradation, are identified to promote the aging of MPs [16]. In a study conducted on agricultural farmland soil, ARG abundance on MPs was found to be dependent on MP size, weathering, and years of cultivation [57].

### Adsorption of Co-Contaminants

**Figure 3:**
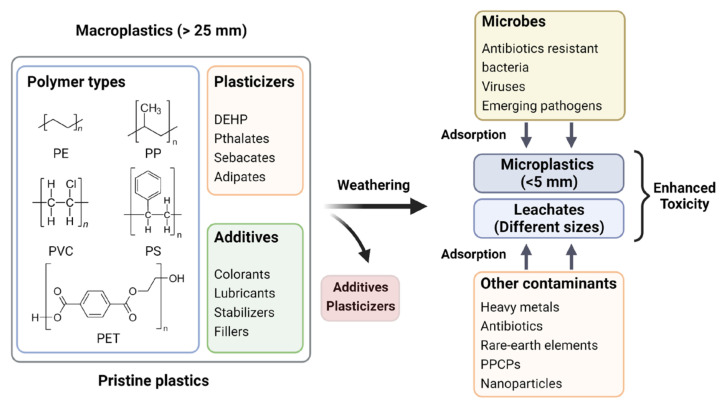
Increased toxicity of MPs/MP-leachates in agroecosystem upon aging and gradual adsorption/accumulation of co-contaminants.

Though MPs are classified as emerging pollutants, they may not be hazardous per se to living beings in their native chemical compositions in a pristine microenvironment. However, their omnipresence in the environment is a matter of concern owing to their role as a vector for several hazardous agents, including heavy metals [63], rare earth elements [64], pharmaceuticals (antibiotics) [65,66], personal care products [67,68], and a variety of other emerging contaminants of concern including microbes (antibiotic-resistant bacteria, viruses, etc.) [69]. Figure 3 shows a pictorial representation of such a long-term natural process amplifying the toxicity of MPs in agroecosystems. The vector hypothesis has led to forecast MPs contamination in the food chain, which poses a hazard to the living world by increasing the risk of outbreaks of food-borne diseases [70]. The extent of adsorption and desorption of contaminants on MPs depend on the properties of both MPs and contaminants, such as hydrophobicity, surface area, and surface functional groups [11,71] and the MPs frequently tend to selectively accumulate certain contaminants, resulting in very high local concentration even though the concentrations in the environment are below detection limits [72]. The interactions between MPs and other pollutants are driven by weak intermolecular forces such as hydrogen bonding, van der Waals force, electrostatic interaction [73], pore filling, and π-π interactions [74,75]. The pH [76,77], viscosity, salinity [78], ionic strength of the media, presence or absence of dissolved organic matter [79], diffusion of intraparticle and films, photodegradation, and mechanical stress, are also known to influence the adsorption of contaminants on MPs [80]. The mobility of pollutants in water or soil matrix increases to varying degrees depending on the amount of adsorption of contaminants onto MPs [81]. Therefore, pollutant-adsorbed MPs may cause severe disruptions in the agroecosystems by increasing the risks of ecotoxicity and bioaccumulation of toxic agents, having deleterious effects on living systems in the environment [82].

## 5. Rapid Detection, Identification, and Quantification of MPs from Environmental Matrices

The currently available methods for separating and detecting MNPs from a complex matrix such as soil have several limitations, restricting them mainly to qualitative enumeration (for MPs) or estimation of total mass (for NPs). Scientists worldwide are actively engaged in developing standardized protocols for separating MPs from the soil matrix and their identification [83]. Techniques currently adopted by researchers for the isolation of MNPs (of different types/states/nature) from different complex environmental matrices are summarized in Table 3. To detect and quantify MPs, a variety of techniques are currently employed alone or in combination, which includes infra-red spectroscopy (ATR-FTIR, micro-FTIR), Raman spectroscopy, electron microscopy, and mass-spectrometry coupled with gas chromatography.

### 5.1. Methodological Challenges

MPs are heterogeneous in nature; they can be formed by different polymers and present with various densities, shapes, and structures. Additionally, they often contain additives and are found in different aging states. These properties of MPs make their extraction, detection and quantification in complex matrices challenging. The smaller size of NPs further prevents their sampling and efficient detection from any complex matrix by techniques commonly employed for MPs detection. Furthermore, the optical detection of NPs is diffraction limited, impeding their analysis below the size of 300 nm. Moreover, when compared with MPs, the smaller size of the NPs poses further restrictions in the availability of reference materials [101]. Sampling and extraction of MPs from a complex matrix such as agricultural soil is commonly carried out by digestion of biological materials by Fenton oxidation treatment, followed by centrifugation at 3700 rpm for 10 min, and finally visualization of extracted MPs is performed by Nile red staining and fluorescence microscopy [84]. A number of other techniques are described for MNP extraction, including sieving [86], filtration and dialysis [87], ultrasonication [102], pressurized solvent extraction (PSE) [90,92], and chemical digestion of organic materials [83,103]. Each of these extraction techniques has their strengths and limitations, and therefore, the selection of an optimal method for a specific sample should be guided by the sample characteristics. Sample preparation and handling (prevention of contamination) is a huge challenge for reliable detection of MPs. Variation in sample preparation influences the recovery of MPs after extraction from complex matrices. Recent study has compared different sample preparation methods, highlighting their effect on average recoveries of MPs from water samples [83]. A novel Small Volume Glass Separator and the related standard operating procedures have been developed to overcome the lack of consistency in MPs recovery [83]. The detection and analysis of extracted MNPs are commonly performed by pyrolysis gas chromatography-mass spectrometry (py-GC-MS), and Fourier transform infrared spectroscopy (FTIR)/Raman spectroscopy [85,87,90,94,104]. These techniques have greatly improved the detection limits of MPs and NPs; even with these sensitive techniques, residual organic matter/minerals can cause difficulty in the detection; thus, a suitable extraction plays a critical role in the analysis [95].

### 5.2. Recent Developments in Analytical Methods for MNP Detection

In recent years, a number of new techniques, alone or in combination with existing techniques, are increasingly used for MNP analysis. Some of these techniques are specialized ATR-FTIR with hyperspectral imaging, GC-ToF with thermo-gravitimetric analysis [93], Laser directed infrared (LDIR) [31,103], and micro-FTIR [92], which are often coupled with existing visualization techniques such as scanning/transmission electron microscopy (SEM/TEM-EDS) [88], stereomicroscopy [86], and X-ray photoelectron microscopy [105]. Each of these techniques is limited by a set of advantages and disadvantages. For example, Raman imaging being insensitive to water provides a greater resolution in aquatic sample visualization, but there can be fluorescence interference from background organic–inorganic residues and additives. There is also a risk of overestimation or underestimation when MNPs form aggregates with natural particles. Moreover, setting up parameters such as laser wavelength and magnification can be tricky [98,101]. Using currently available technologies, reliable detection of each individual MPs sized 4–5 µm are possible; however, only mass concentration can be obtained for NPs of ≤1.0 µm. Some modern techniques utilize UV-Vis method to quantify NPs from different organic matters with non-preferential adsorption of the particles to geothite and resulting in increased specific absorbance [97]; liquid chromatography-mass spectrometry (LC-MS) is often used for detection of MNPs with limit of detection (LOD) up to 2.0 µg/L and 1.2 µg/L for influents and effluents respectively [99]. Ref. [96] used multi-functional, light-powered, and self-propelled MXene-derived γ-Fe_2_O_3_/Pt/TiO_2_ microrobots for NP detection. They can trap NPs via magnetic attraction with their stacks and enable on-site electrochemical detection and screening. The use of a fluorescent molecular rotor for NP detection in radish sprouts and mussel tissues has been performed, paving the way for new perspectives to overcome challenges in this field [100]. Researchers around the world are focusing on developing more automated detection techniques and standardization of already-developed protocols as more sensitive and higher-resolution technologies are needed to meet the current challenges in the detection and analysis of MNPs [106].

## 6. Conclusions

MPs provide a suitable surface for attachment of microbes and the formation of biofilms. The slimy nature of biofilm plays a major role in the subsequent attachment and adsorption of many other pollutants and pathogenic microbes, thereby becoming a hub or hotspot for gene transfer. Such close interactions among microbes, and between microbes and pollutants, greatly influence the resistome, thereby enhancing the spread of AMR. Several other properties of MPs, particularly their chemical composition, surface charge, or size, play an important role in gene transfer efficiency between microbes. Moreover, strongly aged MPs had higher ARG abundance compared to weakly aged ones. In agricultural soil, the persistence of MPs and their interaction with soil microbes also influence soil nutrient balance and crop productivity. This review highlighted several factors that have been studied and reported, specifically focusing on the mechanism of horizontal dissemination of ARGs, changes in resistome profile, and the resultant impact on the agroecosystem. However, this area of research is only in its initial phase, and we strongly believe that several other important factors may be yet to be identified. The existing body of research on the impact of MPs in the ecosystem shows that MPs may enter the food chain in several ways and affect human health. However, current research is still in the stage of exploration. Furthermore, accurate quantification of MPs in complex matrices from environmental samples is impacted by the lack of appropriate techniques and standard protocols for extraction and detection of MNPs. In this review, we summarized the promising recent advancements in the extraction and detection of MNPs. Overall, a deeper insight into MNP contamination in agroecosystems and its impact on different related constituents needs continued and extensive research. It is also crucial that knowledge from these should be utilized to benefit humankind and protect human health by implementing proper control measures for plastic usage in agriculture.

## Figures and Tables

**Table 1:** Major use and application of plastics in agriculture. (adopted from [20]).

**Protective cultivation films:** covering vineyards/orchards direct covering greenhouses and tunnels high and low tunnels mulching nursery films	**Piping, irrigation/drainage:** channel lining drainage pipes drippers irrigation tapes and pipes micro irrigation water reservoir	**Nets:** anti-bird anti-hail nets for olives/nuts harvesting shading wind-breaking
**Other films:** fumigation films silage films	**Packaging:** fertilizers sacks agro-chemical cans/bottles containers tanks for liquid storage crates	**Other:** baling twines baling wraps nursery pots strings and ropes

**Table 2:** A list of representative studies on the interaction of MNPs with microbes leading to the HGT and enhanced AMR.

Type of MPs	Model Bacteria	Research Setup or Test Matrices	Possible Mechanisms	Reference
Polystyrene (PS-MPs/PS-NPs)	*E. coli* DH5α (recipient) plasmid pUC19 (amp^R^)	Transformation of plasmid borne ARG into recipient bacteria *E. coli* DH5α, in co-presence of humic acid or Fe^3+^.	NPs (10–500 mg/L) significantly enhanced the transformation efficiency. NPs induced ROS overproduction, activated SOS response, increased cell membrane permeability, and changed the secretion systems, thereby facilitating the uptake of exogenous DNA by bacteria.	[50]
Polystyrene (PS-MPs)	*E. coli* MG1655 (recipient) *E. coli* DH5α	Effects of Pristine PS-MPs and UV-irradiated PS-MPs on HGT. Differential gene expression analysis of donor bacteria for conjugation regulating genes, recipient bacteria for genes regulating bacterial sensitivity to phage lambda and genes associated with intracellular ROS production	UV-aged MPs enhanced horizontal ARG transfer compared to pristine MPs. Conjugation was the dominant mechanism. MPs interact via hydrophobic attraction and π-π stacking. Aged MPs upregulated genes regulating conjugation, associated with cell permeability, and intracellular ROS production	[30]
Polystyrene (PS) MPs	*E. coli* DH5α (RP4 plasmid) (Donor) *E. coli* K12 MG1655 (recipient)	Effect of MNPs size and concentration on HGT. ROS production, cell membrane permeability and viability assay. Analysis of gene expression upon exposure to MPs, through qPCR	The gene transfer efficiency depends on mating time, ratio of bacteria and size, concentration of MNPs. MNPs increased ROS production and cell membrane permeability of the donor and the recipient. NPs at concentration 100 mg/L led to excessive ROS effecting viability of bacteria.	[33]
Polystyrene (PS) MPs	Phosphorus removing bacteria	Lab-scale batch reactors (working volume 1 L) operated at 20 °C, for 30 days containing different concentrations of MPs. After of MPs exposure, microbial communities explored by high-throughput sequencing. Quantification of ARGs also performed.	MP/NPs promoted the propagation of ARGs in biological phosphorus removal system. Microbial community shift rather than HGT was the main factor promoting ARG propagation under MP/NP pressure.	[49]
Polyvinyl chloride (PVC), Polyamide (PA), Polyethylene (PE), Polystyrene (PS) MP beads (30 μm)	Soil microbial communities	Soil microcosm experiments, to study the effects of manure, temperature, and moisture on microbiome and antibiotic resistome. Amplicon sequencing and HT-qPCR to detect ARGs, and the bacterial community of plastispheres. Field experiment with metagenomic sequencing to study antibiotic resistome of different plastispheres.	The MPs select for microbial communities in the plastisphere (depending on type and size), and these microbiota are involved in a variety of ecological processes in the soil ecosystem. Plastispheres represents a hotspot of increased potential for the HGT.	[23]
Polystyrene (PS MPs (diameter 3 mm, height 4 mm).	*E. coli* HB101 (plasmid RP4) (Donor) *E. coli* NK5449 resistant to rifampicin and Nalidixic acid (Recipient)	Two types of representative materials, nanoalumina (nanomaterial, conjugative pili promoter) and FNA (conjugative pili inhibitor), were used to verify effect on HGT. Assay of intracellular ATP concentration, and bacterial colonization in MPs using SEM.	RP4 plasmid promotes bacterial colonization on MPs, promoting biofilm development. Negligible effect of donor bacteria alone, on bacterial colonization. When donors and recipient coexist, intracellular ATP concentration and intracellular energy supply increase, simultaneously increasing the expression level of conjugative pili synthesis genes.	[8]
Low density polyethylene (LDPE), Polypropylene (PP), Polystyrene (PS)	Soil bacteria	Soil microcosm experiment with 100 g soil in sterilized 350 mL glass jar (incubated at 25 °C for 14 days to activate the microbiota). Three types of MPs (sizes 75 and 550 µm) were added at 2% concentration, incubation for 90 days. DNA was extracted from 0.5 g soil after experiment and 0.2 g of extracted MPs from soil, sequenced and aligned against the Comprehensive Antibiotic Resistance Database for ARG identification	Total abundance of different ARGs was significantly higher in the plastispheres compared to the surrounding soil under all treatments. Enrichment of ARGs in the plastisphere (resistome) varied across MP types, but not much on MPs size, which also effected the microbial community structure.	[5]
Polyvinyl chloride (PVC), polyamide (PA), polystyrene (PS), and polyethylene (PE) MPs	Ammonia-oxidizing, Nitrite-oxidizing bacteria	The sequencing batch reactor operated for a total of 84 days with added MPs at different concentration. The microbial community of aerobic granular sludge from reactor and MPs associated biofilm were explored by Illumina Miseq sequencing.	PVC, PA and PS stimulated the secretion of extracellular polymeric substances and ROS species. Shifts of nitrification genes in aerobic granular sludge and on MPs biofilms	[35]
Polyvinyl chloride (PVC) MP pellets (3 mm)	Biofilm	Sterilized MPs, rock, and leaf wrapped into different aggreates, used in bioreactor fed with 5 L river water, incubated for 2 weeks. Biofilms formed on different substrates was investigated using SEM. DNA extracted from biofilms, followed by 16S rRNA gene sequencing and shotgun metagenomics to study the relative abundance of ARGs type.	Biomass of MPs associated biofilm was more than rock biofilm but less than leaf biofilm. MP associated biofilm had distinctive microbial communities structure compared with rock and leaf biofilms. The ARG abundance of biofilm was ~three-fold higher than that of river water, indicating high diversity ARGs enriched by biofilm cultured with water sampled from river.	[27]

**Table 3:** Frequently used methodologies for detecting MPs/NPs from complex environmental matrices.

Targeted Environmental Matrix	Extraction Methods Used	Detection and Quantification Techniques	Type of MPs/NPs Detected	**Detection Limit**	**Reference**
Agricultural soil	Centrifugation	Fluorescence microscopy, Nile red staining, Image processing with Fiji and Python	LDPE, PP, PS, PVC, PET	6–20 mg/µg	[84]
Estuarian muddy sediment	Homogenization, freeze drying	Pyrolysis-GC/MS, FTIR/Raman spectroscopy	PP, PS, PET, PVC	Particles per µg of sample	[85]
Drinking water	Filtering/sieving	Stereomicroscopy, FTIR/Raman spectroscopy	PE, PS, PET, PVC	% of particle recovery	[86]
Agricultural soil	Ultrasonication, filtration	ATR-FTIR, LDIR	PE, PP, PVC, PA, PTFE	10^5^ particles per kg soil	[31]
Water (pure water and sea water)	Filtration, dialysis	TEM, DLS, SERS	PS, PP, PE	40 µg/mL (100 nm sized)	[87]
Organic clay colloids and humic acid	Asymmetric flow field-flow fractionation (AF4)	AF4-TOC (Total organic carbon), FLD, dRI, UV, Nile red staining	PS	Comparison of different methods	[88]
Sediment	Density separation JAMSTEC microplastic-sediment separator (JAMSS) unit	Japan Agency for Marine-Earth Science and Technology (JAMSTEC)	PE, PP, PS, PVC, PET	94–98% of MPs < 1000 µm	[89]
Biosolids	Pressurized liquid extraction (PLE)	Double shot pyrolysis GC/MS	PC, PE, PS, PP, PET, PVC, PMMA	2.8–6.6 mg/g	[90]
Surface water and sediment	Density separation-MPSS	Pyrolysis-GC/MS, Hyperspectral FTIR imaging spectroscopy	PE, PEST, PP, PS, PVC, PC, PMMA, PUR, PA	Mass of MPs in µg m^−3^	[91]
Marine sponge	Pressurized solvent extraction (PSE)	Pyrolysis-GC/MS, Micro-FTIR	PS, PP, PE, PVC, PC, PL, PA	6.6–30.2 ng/g	[92]
Powdered plastic	Filtration	Pyrolysis GC-ToF, Thermo-gravitimetric analysis, GC-ToF	PP, PS, PVC	<50 µg/L	[93]
Aquatic environment		Photo-induced force microscopy (PiFM), Infrared spectroscopy	PET	Count of NP size ~20 nm	[94]
Soil	Density gradient separation	Transmission-type terahertz time-domain spectrometer and NIR hyperspectral imaging system	PS, PVC	1.12% (tetrahertz) 3.34% (NIR)	[95]
Water	self-propelled light-powered MXene-derived γ-Fe_2_O_3_/Pt/TiO_2_ microrobots	SEM, Electrochemical impedance spectroscopy (EIS), XPS, NTA	NPs	10^6^ NPs per mL	[96]
Natural organic matter	Absorptive fractionation	UV-Vis	PSNPs	7.4 mgC/L	[97]
Underwater	On-site detection (NO extraction)	DEP-ACEO-Raman tweezer (DART) (Dieletrophoresis and AC electro-osmosis)	NPs (PS, PMMA)	1.17 μg·L^−1^	[98]
Waste-water	Drying	PET depolymerization, LC-MS	MNPs (PET)	Influent-2.0 µg/L, effluent-1.2 µg/L	[99]
Water, radish seeds, mussels	Drying (vegetal samples), centrifugation, chemical digestion, filtration (animal samples)	Fluorescent Molecular Rotor (FMR)	PS NPs	475–563 µg/L in pure water	[100]

## Data Availability

This review used information from published peer-reviewed articles. All data and findings reported in this paper can be found in the respective references.
